# 1,1′-{(Hexane-1,6-di­yl)bis­[(aza­niumylyl­idene)methanylyl­idene]}bis­(naphthalen-2-olate)

**DOI:** 10.1107/S2056989014027236

**Published:** 2015-01-01

**Authors:** Kamel Ouari, Sabrina Bendia, Moufida Merzougui, Corinne Bailly

**Affiliations:** a Laboratoire d’Electrochimie, d’Ingénierie Moléculaire et de Catalyse Redox, Faculty of Technology, University of Sétif-1, 19000 Sétif, Algeria; b Service de Radiocristallographie, Institut de Chimie de Strasbourg, UMR 7177, CNRS–Unistra, 1 rue Blaise Pascal, Strasbourg 67008, France

**Keywords:** crystal structure, 1,6-di­amino­hexa­ne, 2-hy­droxy-1-naphthaldehyde, hydrogen bonding, elemental analysis.

## Abstract

The whole molecule of the title Schiff base compound, C_28_H_28_N_2_O_2_, is generated by inversion symmetry. It is formed from two units of *ortho*-hy­droxy­naphthaldehyde bridged with 1,6-di­amino­hexane. The N atoms are protonated and, thus, the structure is a bis-zwitterionic compound in the solid state. The zwitterion shows strong intra­molecular N—H⋯O hydrogen bonds between the iminium N and the naphthaleno­late O atoms.

## Related literature   

For the synthesis of similar compounds, see: Ramos Silva *et al.* (2009 ▸); Li *et al.* (2007 ▸); Zhu *et al.* (2006 ▸); Sampath Kumar *et al.* (2010 ▸); Bhattacharjee *et al.* (2012 ▸). For their applications, see: Ourari *et al.* (2006 ▸, 2008 ▸); Ouari *et al.* (2010 ▸, 2015 ▸). For related crystal structures, see: Yuan & Li (2013 ▸); Paul & Kubicki (2009 ▸). For the biological activity of Schiff bases, see: Zayed *et al.* (2015 ▸); Abou-Hussein & Linert (2014 ▸); Sadeek *et al.* (2013 ▸).
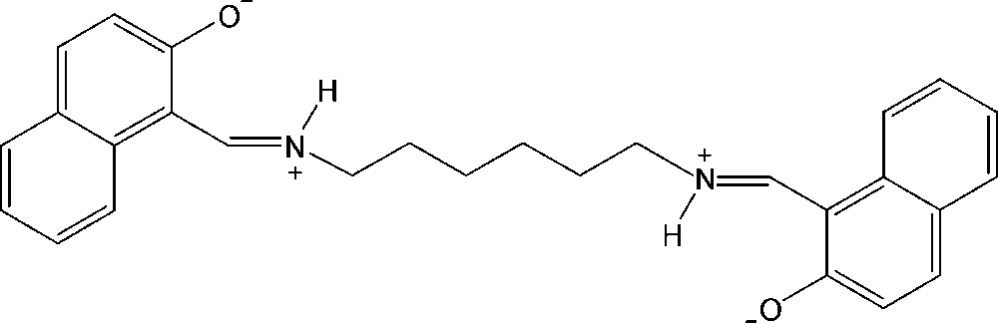


## Experimental   

### Crystal data  

C_28_H_28_N_2_O_2_
*M* *_r_* = 424.52
Orthorhombic, 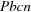

*a* = 23.722 (1) Å
*b* = 8.8117 (3) Å
*c* = 10.3903 (5) Å
*V* = 2171.90 (16) Å^3^
*Z* = 4
Mo *K*α radiation
μ = 0.08 mm^−1^
*T* = 173 K
0.36 × 0.16 × 0.08 mm

### Data collection  

Nonius KappaCCD diffractometer
17177 measured reflections
2500 independent reflections
1285 reflections with *I* > 2σ(*I*)
*R*_int_ = 0.082

### Refinement  

*R*[*F* ^2^ > 2σ(*F* ^2^)] = 0.054
*wR*(*F* ^2^) = 0.160
*S* = 0.99
2500 reflections
150 parameters
H atoms treated by a mixture of independent and constrained refinement
Δρ_max_ = 0.23 e Å^−3^
Δρ_min_ = −0.28 e Å^−3^

### 

Data collection: *COLLECT* (Nonius, 1998 ▸); cell refinement: *DENZO* (Otwinowski & Minor, 1997 ▸); data reduction: *SCALEPACK* (Otwinowski & Minor, 1997 ▸); program(s) used to solve structure: *SHELXS2013* (Sheldrick, 2008 ▸); program(s) used to refine structure: *SHELXL2013* (Sheldrick, 200); molecular graphics: *PLATON* (Spek, 2009 ▸); software used to prepare material for publication: *SHELXL2013*.

## Supplementary Material

Crystal structure: contains datablock(s) I. DOI: 10.1107/S2056989014027236/mw2128sup1.cif

Structure factors: contains datablock(s) I. DOI: 10.1107/S2056989014027236/mw2128Isup2.hkl

**Fig. 1.**
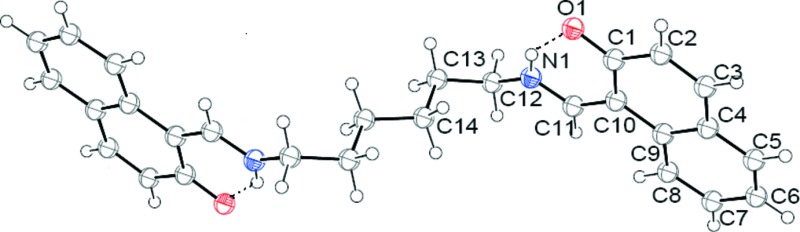
The molecular geometry of the title compound with displacement ellipsoids drawn at the 50% probability level. H atoms are represented as small spheres of arbitrary radii. Hydrogen bonds are shown as dashed lines. Only the non-H atoms of the asymmetric unit are labelled.

**Fig. 2.**
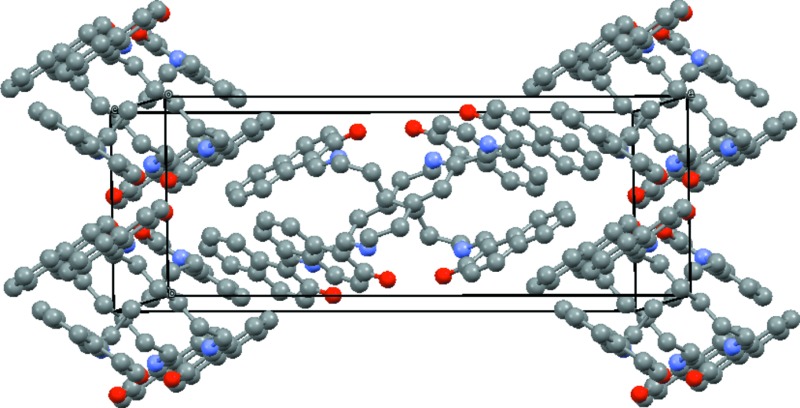
Crystal packing of the title compound viewed along the c axis.

. DOI: 10.1107/S2056989014027236/mw2128fig1.tif
The mol­ecular geometry of the title compound with displacement ellipsoids drawn at the 50% probability level. H atoms are represented as small spheres of arbitrary radii. Hydrogen bonds are shown as dashed lines. Only the non-H atoms of the asymmetric unit are labelled.

c . DOI: 10.1107/S2056989014027236/mw2128fig2.tif
Crystal packing of the title compound viewed along the *c* axis.

CCDC reference: 1032693

Additional supporting information:  crystallographic information; 3D view; checkCIF report

## Figures and Tables

**Table 1:** Hydrogen-bond geometry (, )

*D*H*A*	*D*H	H*A*	*D* *A*	*D*H*A*
N1H1*N*O1	0.99(3)	1.74(3)	2.587(2)	141(2)

## supplementary crystallographic information

### S1. Experimental

The Schiff base ligand was prepared in 67% yield by condensation between 58 mg (0.5 mmole) of 1,6-di­amino­hexane and 172 mg (1 mmole) of 2-hy­droxy-1-naphthaldehyde in methanol (12 mL). The mixture was refluxed and stirred under a nitro­gen atmosphere for 3 hours. The precipitate obtained was filtered, washed with methanol and di­ethyl ether and dried in vacuum overnight. The product was recrystallized from di­methyl sulfoxide at room temperature over a period of a week. The yellow, single crystals of C_28_H_28_O_2_N_2_ obtained were of X-ray quality. Elemental analysis: calculated for C_28_H_28_O_2_N_2_: C 79.20, H 6.65, N 6.60%; found: C 78.84, H 6.63, N 6.78%.

### S1.1. Refinement

The iminium H atom was located from a difference Fourier map and refined isotropically. C-bound H atoms were included in calculated positions and treated as riding atoms: C—H = 0.95 Å (CH) or 0.99 Å (CH_2_) with Uiso(H) = 1.2Ueq(C—H_aromatics_). .

### S2. Results and discussion

The synthesis of the Schiff base is similar to those described in the literature (Ramos Silva *et al.*, 2009; Li *et al.*, 2007; Zhu *et al.*, 2006; Sampath Kumar *et al.*, 2010; Bhattacharjee *et al.*, 2012). These ligands are also currently applied in coordination chemistry for the synthesis of Schiff base complexes of transition metals (Ouari *et al.*, 2015; Ouari *et al.*, 2010; Ourari *et al.*, 2008; Ourari *et al.*, 2006). Compounds of the type of the title molecule possess diverse biological properties such as anti-anxiety, anti-depressant (Zayed *et al.*, 2015) and anti-tumor activities as well as anti­bacterial and fungicidal properties (Abou-Hussein *et al.*, 2014; Sadeek *et al.*, 2013). We report here the synthesis of title compound and its crystal structure.

A perspective view of the title molecule, which has crystallographically- imposed centrosymmetry, is shown in Fig. 1. The intra­molecular N1—H1N···O1 hydrogen bond forces the O1–C1–C10–C11–N1 unit into near planarity (rms deviation 0.005 Å) with the consequence that the naphthalene portion is nearly co-planar with it (dihedral angle 1.20 (8)°).

### Crystal data

**Table:**

C_28_H_28_N_2_O_2_	*D*_x_ = 1.298 Mg m^−^^3^
*M**_r_* = 424.52	Mo *K*α radiation, λ = 0.71073 Å
Orthorhombic, *P**b**c**n*	Cell parameters from 8957 reflections
*a* = 23.722 (1) Å	θ = 1.0–27.5°
*b* = 8.8117 (3) Å	µ = 0.08 mm^−^^1^
*c* = 10.3903 (5) Å	*T* = 173 K
*V* = 2171.90 (16) Å^3^	Prism, yellow
*Z* = 4	0.36 × 0.16 × 0.08 mm
*F*(000) = 904	

### Data collection

**Table:**

Nonius KappaCCD diffractometer	1285 reflections with *I* > 2σ(*I*)
Radiation source: sealed tube	*R*_int_ = 0.082
Graphite monochromator	θ_max_ = 27.5°, θ_min_ = 2.5°
phi and ω scans	*h* = −25→30
17177 measured reflections	*k* = −10→11
2500 independent reflections	*l* = −13→12

### Refinement

**Table:**

Refinement on *F*^2^	Hydrogen site location: mixed
Least-squares matrix: full	H atoms treated by a mixture of independent and constrained refinement
*R*[*F*^2^ > 2σ(*F*^2^)] = 0.054	*w* = 1/[σ^2^(*F*_o_^2^) + (0.0842*P*)^2^] where *P* = (*F*_o_^2^ + 2*F*_c_^2^)/3
*wR*(*F*^2^) = 0.160	(Δ/σ)_max_ < 0.001
*S* = 0.99	Δρ_max_ = 0.23 e Å^−^^3^
2500 reflections	Δρ_min_ = −0.28 e Å^−^^3^
150 parameters	Extinction correction: *SHELXL2013* (Sheldrick, 2008), Fc^*^=kFc[1+0.001xFc^2^λ^3^/sin(2θ)]^-1/4^
0 restraints	Extinction coefficient: 0.0047 (14)

### Special details

**Table:**

Geometry. All e.s.d.'s (except the e.s.d. in the dihedral angle between two l.s. planes) are estimated using the full covariance matrix. The cell e.s.d.'s are taken into account individually in the estimation of e.s.d.'s in distances, angles and torsion angles; correlations between e.s.d.'s in cell parameters are only used when they are defined by crystal symmetry. An approximate (isotropic) treatment of cell e.s.d.'s is used for estimating e.s.d.'s involving l.s. planes.

### Fractional atomic coordinates and isotropic or equivalent isotropic displacement parameters (Å^2^)

**Table:**

	*x*	*y*	*z*	*U*_iso_*/*U*_eq_	
C1	0.42177 (9)	0.1531 (2)	0.9435 (2)	0.0314 (5)	
C2	0.42703 (9)	0.1083 (2)	1.0765 (2)	0.0336 (5)	
H2	0.4575	0.0451	1.1016	0.040*	
C3	0.38971 (9)	0.1541 (2)	1.1655 (2)	0.0351 (5)	
H3	0.3951	0.1236	1.2524	0.042*	
C4	0.34205 (8)	0.2473 (2)	1.1344 (2)	0.0319 (5)	
C5	0.30246 (9)	0.2899 (2)	1.2282 (2)	0.0389 (6)	
H5	0.3080	0.2583	1.3147	0.047*	
C6	0.25612 (9)	0.3759 (2)	1.1985 (2)	0.0411 (6)	
H6	0.2297	0.4032	1.2631	0.049*	
C7	0.24870 (9)	0.4224 (2)	1.0717 (2)	0.0416 (6)	
H7	0.2167	0.4820	1.0497	0.050*	
C8	0.28662 (9)	0.3839 (2)	0.9781 (2)	0.0359 (5)	
H8	0.2806	0.4182	0.8926	0.043*	
C9	0.33434 (8)	0.2945 (2)	1.0054 (2)	0.0294 (5)	
C10	0.37521 (8)	0.24816 (18)	0.90863 (19)	0.0283 (5)	
C11	0.36924 (9)	0.2932 (2)	0.7794 (2)	0.0311 (5)	
H11	0.3382	0.3570	0.7587	0.037*	
C12	0.39177 (9)	0.2937 (2)	0.55205 (19)	0.0372 (6)	
H12A	0.3640	0.3775	0.5496	0.045*	
H12B	0.3747	0.2051	0.5084	0.045*	
C13	0.44381 (9)	0.3416 (2)	0.4786 (2)	0.0369 (5)	
H13A	0.4324	0.3750	0.3914	0.044*	
H13B	0.4689	0.2526	0.4687	0.044*	
C14	0.47653 (8)	0.4686 (2)	0.54272 (19)	0.0370 (6)	
H14A	0.4932	0.4303	0.6238	0.044*	
H14B	0.4502	0.5518	0.5650	0.044*	
N1	0.40291 (8)	0.25438 (18)	0.68565 (17)	0.0333 (5)	
O1	0.45832 (6)	0.10907 (16)	0.86112 (13)	0.0403 (4)	
H1N	0.4349 (11)	0.195 (2)	0.720 (2)	0.064 (7)*	

### Atomic displacement parameters (Å^2^)

**Table:**

	*U*^11^	*U*^22^	*U*^33^	*U*^12^	*U*^13^	*U*^23^
C1	0.0307 (12)	0.0318 (11)	0.0318 (13)	−0.0016 (8)	0.0006 (10)	−0.0004 (9)
C2	0.0348 (12)	0.0326 (10)	0.0335 (13)	0.0006 (9)	−0.0050 (10)	0.0047 (9)
C3	0.0394 (14)	0.0371 (11)	0.0289 (12)	−0.0057 (9)	−0.0019 (10)	0.0040 (9)
C4	0.0311 (12)	0.0321 (10)	0.0325 (13)	−0.0056 (8)	−0.0003 (10)	−0.0021 (9)
C5	0.0399 (14)	0.0449 (13)	0.0319 (13)	−0.0090 (10)	0.0026 (11)	−0.0056 (9)
C6	0.0307 (13)	0.0495 (13)	0.0431 (15)	−0.0056 (10)	0.0073 (11)	−0.0162 (10)
C7	0.0332 (13)	0.0438 (12)	0.0479 (15)	0.0008 (9)	0.0003 (11)	−0.0105 (11)
C8	0.0347 (13)	0.0363 (11)	0.0367 (13)	0.0009 (9)	−0.0002 (10)	−0.0019 (9)
C9	0.0290 (12)	0.0284 (10)	0.0308 (12)	−0.0039 (8)	−0.0008 (9)	−0.0017 (8)
C10	0.0277 (11)	0.0299 (10)	0.0275 (12)	−0.0009 (8)	−0.0026 (9)	0.0014 (8)
C11	0.0289 (12)	0.0301 (10)	0.0342 (13)	−0.0002 (8)	−0.0012 (10)	−0.0004 (9)
C12	0.0397 (13)	0.0426 (12)	0.0293 (13)	−0.0013 (9)	−0.0026 (10)	0.0060 (9)
C13	0.0398 (13)	0.0430 (12)	0.0281 (12)	0.0014 (10)	0.0010 (10)	0.0041 (9)
C14	0.0413 (13)	0.0409 (11)	0.0288 (13)	0.0008 (10)	0.0005 (10)	0.0027 (9)
N1	0.0349 (10)	0.0380 (10)	0.0271 (11)	0.0018 (8)	0.0010 (9)	0.0042 (7)
O1	0.0383 (9)	0.0474 (8)	0.0352 (9)	0.0120 (7)	0.0035 (7)	0.0006 (7)

### Geometric parameters (Å, º)

**Table:**

C1—O1	1.279 (2)	C8—H8	0.9500
C1—C10	1.433 (3)	C9—C10	1.455 (3)
C1—C2	1.442 (3)	C10—C11	1.408 (3)
C2—C3	1.343 (3)	C11—N1	1.305 (2)
C2—H2	0.9500	C11—H11	0.9500
C3—C4	1.434 (3)	C12—N1	1.455 (2)
C3—H3	0.9500	C12—C13	1.511 (3)
C4—C5	1.405 (3)	C12—H12A	0.9900
C4—C9	1.416 (3)	C12—H12B	0.9900
C5—C6	1.370 (3)	C13—C14	1.516 (3)
C5—H5	0.9500	C13—H13A	0.9900
C6—C7	1.391 (3)	C13—H13B	0.9900
C6—H6	0.9500	C14—C14^i^	1.528 (4)
C7—C8	1.367 (3)	C14—H14A	0.9900
C7—H7	0.9500	C14—H14B	0.9900
C8—C9	1.408 (3)	N1—H1N	0.99 (3)
			
O1—C1—C10	122.03 (18)	C11—C10—C1	118.94 (18)
O1—C1—C2	119.99 (18)	C11—C10—C9	120.86 (18)
C10—C1—C2	117.97 (19)	C1—C10—C9	120.19 (18)
C3—C2—C1	121.40 (19)	N1—C11—C10	125.19 (19)
C3—C2—H2	119.3	N1—C11—H11	117.4
C1—C2—H2	119.3	C10—C11—H11	117.4
C2—C3—C4	122.44 (19)	N1—C12—C13	113.56 (17)
C2—C3—H3	118.8	N1—C12—H12A	108.9
C4—C3—H3	118.8	C13—C12—H12A	108.9
C5—C4—C9	119.48 (19)	N1—C12—H12B	108.9
C5—C4—C3	121.6 (2)	C13—C12—H12B	108.9
C9—C4—C3	118.92 (18)	H12A—C12—H12B	107.7
C6—C5—C4	121.9 (2)	C12—C13—C14	113.75 (18)
C6—C5—H5	119.1	C12—C13—H13A	108.8
C4—C5—H5	119.1	C14—C13—H13A	108.8
C5—C6—C7	118.5 (2)	C12—C13—H13B	108.8
C5—C6—H6	120.7	C14—C13—H13B	108.8
C7—C6—H6	120.7	H13A—C13—H13B	107.7
C8—C7—C6	121.1 (2)	C13—C14—C14^i^	112.7 (2)
C8—C7—H7	119.4	C13—C14—H14A	109.1
C6—C7—H7	119.4	C14^i^—C14—H14A	109.1
C7—C8—C9	121.7 (2)	C13—C14—H14B	109.1
C7—C8—H8	119.2	C14^i^—C14—H14B	109.1
C9—C8—H8	119.2	H14A—C14—H14B	107.8
C8—C9—C4	117.29 (18)	C11—N1—C12	122.55 (19)
C8—C9—C10	123.64 (18)	C11—N1—H1N	109.7 (14)
C4—C9—C10	119.06 (17)	C12—N1—H1N	127.7 (14)
			
O1—C1—C2—C3	179.21 (17)	C3—C4—C9—C10	−0.5 (3)
C10—C1—C2—C3	−0.1 (3)	O1—C1—C10—C11	0.7 (3)
C1—C2—C3—C4	1.1 (3)	C2—C1—C10—C11	180.00 (16)
C2—C3—C4—C5	177.89 (18)	O1—C1—C10—C9	179.50 (17)
C2—C3—C4—C9	−0.8 (3)	C2—C1—C10—C9	−1.2 (3)
C9—C4—C5—C6	0.3 (3)	C8—C9—C10—C11	0.8 (3)
C3—C4—C5—C6	−178.41 (18)	C4—C9—C10—C11	−179.71 (16)
C4—C5—C6—C7	−0.4 (3)	C8—C9—C10—C1	−178.02 (17)
C5—C6—C7—C8	−0.1 (3)	C4—C9—C10—C1	1.5 (3)
C6—C7—C8—C9	0.7 (3)	C1—C10—C11—N1	0.5 (3)
C7—C8—C9—C4	−0.8 (3)	C9—C10—C11—N1	−178.29 (17)
C7—C8—C9—C10	178.74 (17)	N1—C12—C13—C14	−53.7 (2)
C5—C4—C9—C8	0.3 (3)	C12—C13—C14—C14^i^	−171.2 (2)
C3—C4—C9—C8	179.01 (16)	C10—C11—N1—C12	174.49 (16)
C5—C4—C9—C10	−179.23 (16)	C13—C12—N1—C11	139.58 (19)

Symmetry code: (i) −*x*+1, −*y*+1, −*z*+1.

### Hydrogen-bond geometry (Å, º)

**Table:**

*D*—H···*A*	*D*—H	H···*A*	*D*···*A*	*D*—H···*A*
N1—H1*N*···O1	0.99 (3)	1.74 (3)	2.587 (2)	141 (2)
