# Supplementary figures and images for: 1,1′-{(Hexane-1,6-di­yl)bis­[(aza­niumylyl­idene)methanylyl­idene]}bis­(naphthalen-2-olate)
Source: Acta Crystallogr E Crystallogr Commun. 2015 Jan 1;71(Pt 1):o51–2. doi: 10.1107/S2056989014027236 (PMC4331877; doi:10.1107/S2056989014027236)

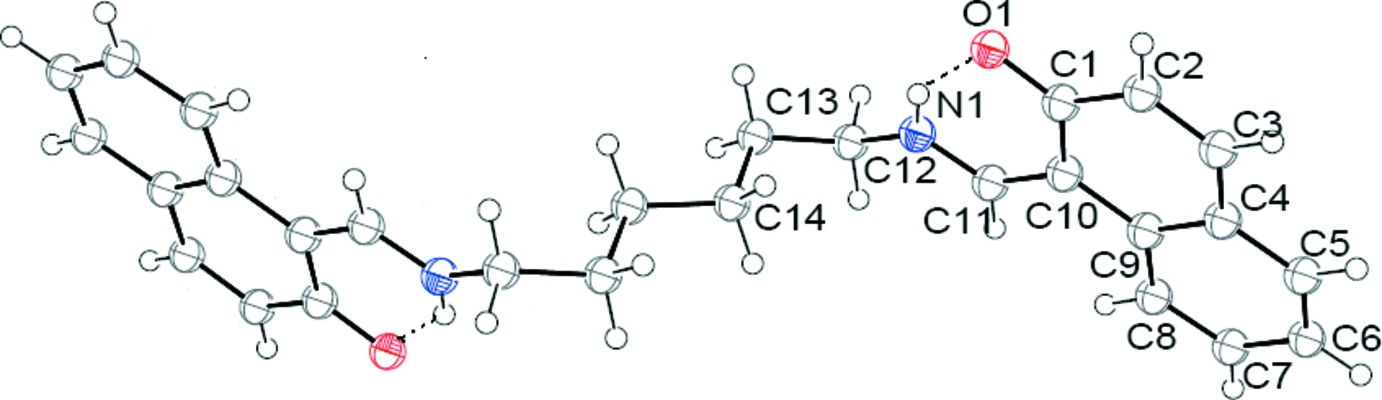

Supplement: Supplementary file 3 [file e-71-00o51-fig1.tif]

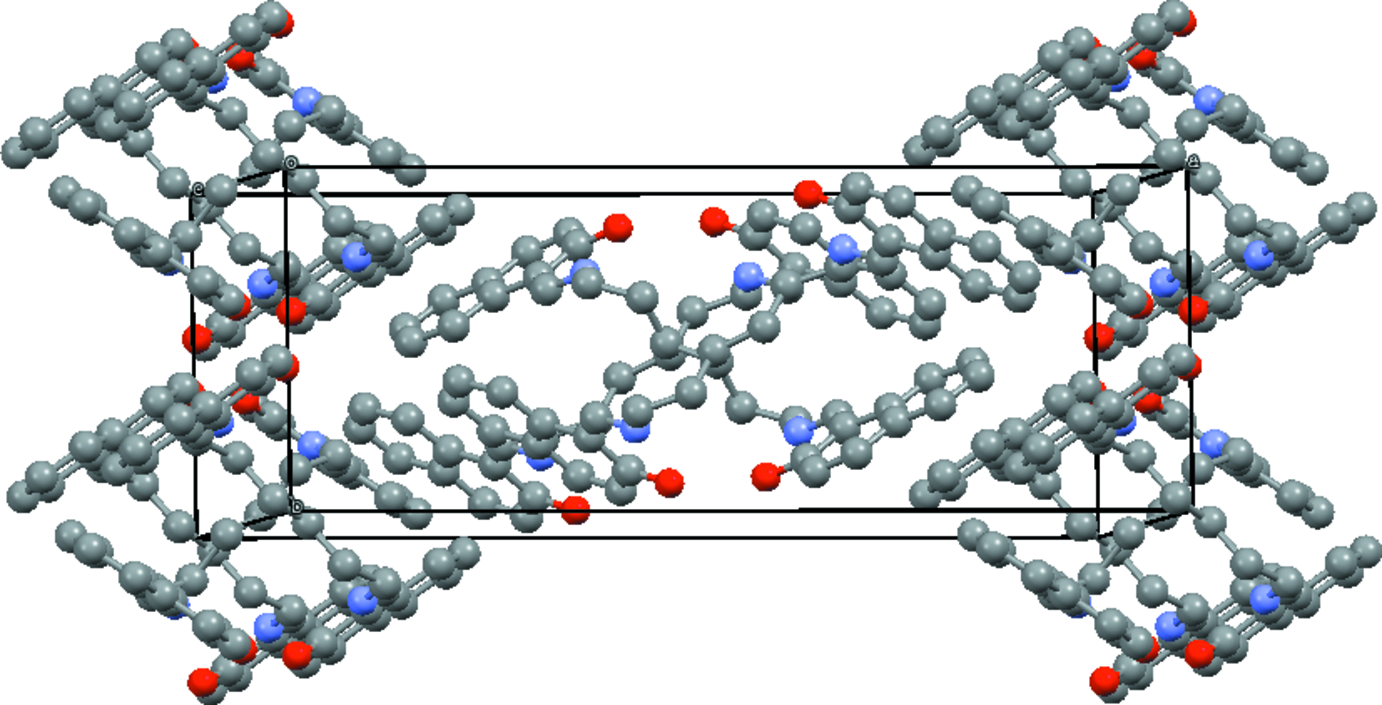

Supplement: Supplementary file 4 [file e-71-00o51-fig2.tif]
